# Coupling a single electron on superfluid helium to a superconducting resonator

**DOI:** 10.1038/s41467-019-13335-7

**Published:** 2019-11-22

**Authors:** Gerwin Koolstra, Ge Yang, David I. Schuster

**Affiliations:** The James Franck Institute and Department of Physics, University of Chicago, Chicago, IL 60637 USA

**Keywords:** Quantum fluids and solids, Superconducting devices, Quantum physics

## Abstract

Electrons on helium form a unique two-dimensional system on the interface of liquid helium and vacuum. A small number of trapped electrons on helium exhibits strong interactions in the absence of disorder, and can be used as a qubit. Trapped electrons typically have orbital frequencies in the microwave regime and can therefore be integrated with circuit quantum electrodynamics (cQED), which studies light–matter interactions using microwave photons. Here, we experimentally realize a cQED platform with the orbitals of single electrons on helium. We deterministically trap one to four electrons in a dot integrated with a microwave resonator, allowing us to study the electrons’ response to microwaves. Furthermore, we find a single-electron-photon coupling strength of $g/2\pi =4.8\pm 0.3$ MHz, greatly exceeding the resonator linewidth $\kappa /2\pi =0.5$ MHz. These results pave the way towards microwave studies of Wigner molecules and coherent control of the orbital and spin state of a single electron on helium.

## Abstract (web summary)

Electrons on the surface of helium have strong interactions with each other but weak coupling to dissipation mechanisms, providing opportunities for many-body physics and storing quantum information. Here the authors demonstrate a circuit QED platform for manipulating and probing few-electron clusters.

## Introduction

Electrons are bound to liquid helium by their induced image charge just below the surface^1^. The orbital state of such electrons consists of the motion parallel to the helium surface and becomes quantized when electrons are trapped in an electrostatic potential. Since the electron-phonon coupling in helium is small compared with semiconductors, this motion is expected to have low dissipation, making the orbital state an attractive candidate for a long-lived electron-on-helium quantum bit^2–6^. In addition, by adding a magnetic field gradient from a micro-magnet^7^, the orbital state offers a path toward the electron spin state^6,8–11^. Since the orbital frequency of electrons on helium is in the microwave regime, and electrons can couple strongly to microwave photons^2,12–14^, cQED can play a unique role in the detection and manipulation of the orbital state.

A small ensemble of electrons on helium behaves differently from other confined electron systems, such as semiconductors or atoms, where the electron wavefunctions are delocalized and overlap. On the surface of liquid helium electron interactions dominate^15,16^ and are largely unscreened^17^, which results in strongly correlated electron configurations known as Wigner molecules^18,19^. The symmetry of these molecules changes for each additional electron, which has been observed in charging diagrams of small islands of liquid helium^20,21^ and only recently in ultraclean nanotubes^22,23^. In addition, theory has predicted Wigner molecule configurations and orbital frequencies in various trapping potentials^24–27^. Coupling these small electron clusters to a microwave resonator could allow for spectroscopy of Wigner molecules in the microwave regime, which would provide insight into both the internal molecular structure and the molecule’s environment.

Here we realize the coupling of a single electron and small electron clusters on helium to a microwave cavity, which serves as an electron detector and harbors an electron reservoir. We transfer electrons from the reservoir to a small island where we control the charge with single electron resolution. Furthermore, we observe unique resonator transmission signatures which allow us to identify different-sized electron clusters, and a large single-electron-photon coupling. These results open the door to studies of the Wigner molecule phase, and coherent control of the orbital and spin state of a single electron on helium.

## Results

### An electron-on-helium dot integrated with cQED

**Fig. 1:**
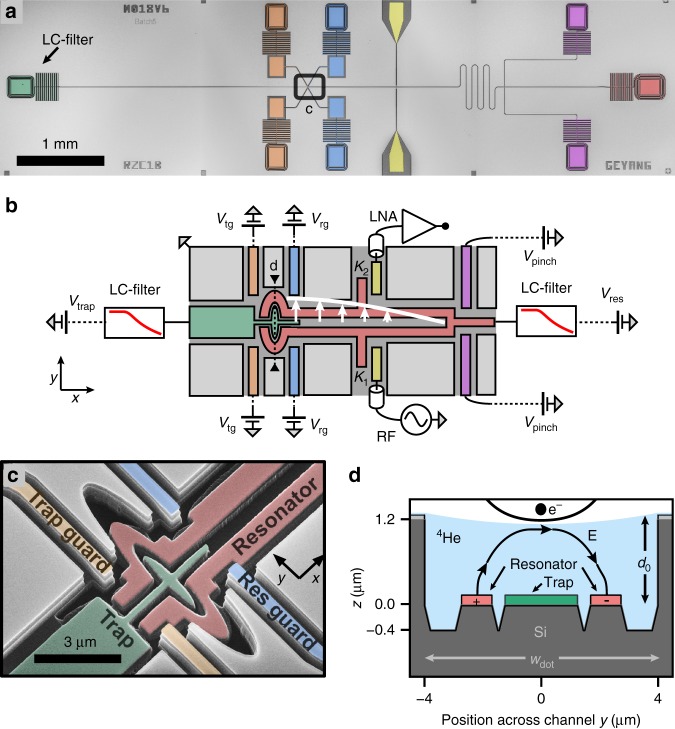
An electron-on-helium dot **a** Optical micrograph and **b** schematic of the device. The resonator (red) can be probed with microwaves via coplanar waveguides (yellow) that couple (decay rates ${\kappa }_{1,2}$) to the microwave resonator. The white arrows show the electric field of the $\lambda /4$ microwave mode at the center of the channel. The transmission is amplified with a low-noise amplifier (LNA). The electrostatic potential for electrons is controlled with additional electrodes, which are all equipped with individual low-pass filters to reject noise at the resonance frequency^28^ (Supplementary Fig. 2). In **b**, we only show these filters for the trap and resonator. **c** Tilted, false-colored scanning electron micrograph of the dot showing the micro-machined silicon substrate. The resonator (red) and trap electrode (green) are located on the bottom of a micro-channel, which lies 1.2 μm below the level of the resonator guards (blue), trap guards (orange) and ground plane. **d** Schematic cross-section of the dot shown in **c**, depicting the resonator center pins and trap electrode submersed in liquid helium. Electrons are trapped on the interface of liquid ^4^He and vacuum by the electrostatic potential (solid black line) generated by electrodes near the dot. The electron orbital state couples to the transverse microwave electric field ${\bf{E}}$ from the resonator.

At the heart of our cQED device lies a superconducting microwave resonator with an integrated electron-on-helium quantum dot (Fig. 1a). Our coplanar stripline resonator consists of two niobium center pins, which are joined at one end (Fig. 1b, c) and are situated below the ground plane at the bottom of a micro-channel (width $w=3.5$ μm, and depth ${d}_{0}\approx$ 1.2 μm). The microwave mode with resonance frequency ${f}_{0}=6.399$ GHz and linewidth ${\kappa }_{{\rm{tot}}}/2\pi =0.4$ MHz has a microwave electric field that is concentrated between the center pins. As liquid ^4^He fills the channel, the helium–vacuum interface is stabilized due to surface tension (see Supplementary Fig. 4), after which liquid helium can serve as a defect-free substrate for electrons (Fig. 1d).

**Fig. 2:**
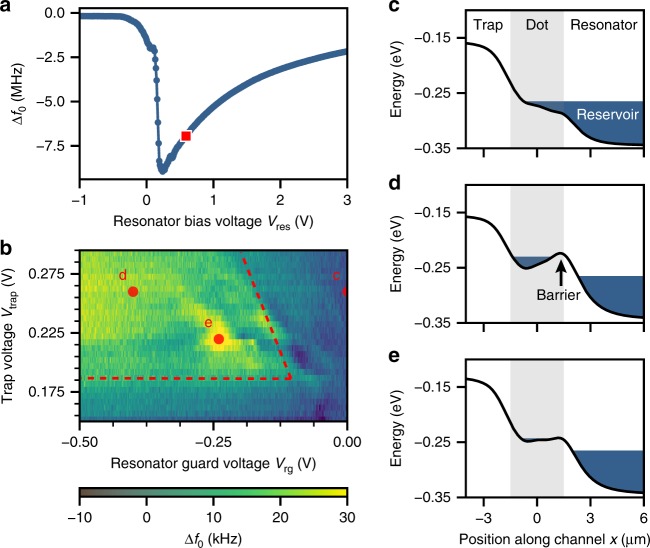
Separating electrons from the reservoir. **a** At $T=25$ mK reservoir electrons are detected through a dispersive resonance frequency shift (blue circles) which depends on ${V}_{{\rm{res}}}$. The jump in $\Delta {f}_{0}$ at ${V}_{{\rm{res}}}\approx 0.18$ V is consistent with electron loss from an ensemble with density $n\approx 9\times 1{0}^{12}$ m^−2^ (see Methods). The data presented hereafter are taken with the resonator bias voltage fixed at 0.6 V, which is marked by a red square. **b** Measured resonance frequency shift while raising a barrier between the dot and reservoir as function of ${V}_{{\rm{trap}}}$. The red dashed line segments mark the border of a region where electrons can be trapped in the dot. The largest $\Delta {f}_{0}$ are expected when the electron orbital frequency approaches ${f}_{0}$. For ${V}_{{\rm{trap}}} \, > \, 0.3$ V electron trapping is unstable, because reservoir electrons can freely flow through the dot onto the trap electrode. **c**–**e** Simulated potential energy along the channel for three different values of ${V}_{{\rm{rg}}}$, ${V}_{{\rm{trap}}}$, marked by the red circles in **b**. Reservoir electrons ($x \, > \, 2$ μm) and electrons in the dot ($-1.5$ μm $< \, x \, < \,1.5$ μm) are represented as a constant energy (blue). Electrons are trapped in the dot in **d** and **e**.

We deposit electrons over the resonator through thermal emission from a tungsten filament situated above the chip (Supplementary Fig. 3), while applying a positive voltage to the resonator DC bias electrode and a negative bias voltage to the filament. We detect the deposited electrons as a dispersive resonance frequency shift that depends strongly on the resonator bias voltage ${V}_{{\rm{res}}}$ (Fig. 2a) and the number of electrons on the resonator^29^. For the experiments presented hereafter, we fix ${V}_{{\rm{res}}}$ at 0.6 V such that electrons on the resonator can be treated as a reservoir with constant electron density. Furthermore, our measurements are performed at $T=25$ mK and low incident microwave power (${n}_{{\rm{ph}}}\approx 5$) such that electrons respond linearly to the resonator’s driving force.

We use the dot in Fig. 1c to isolate individual electrons from the reservoir, which requires fine control over the electrostatic potential. We achieve this using three sets of electrodes near the tip of the resonator where the microwave electric field is strongest. The size of the electrodes near the dot is much larger than in semiconducting quantum dots, because the unscreened electron interaction results in inter-electron distances exceeding 200 nm. With appropriate voltages applied to the electrodes, the smooth electrostatic potential (Fig. 2d,e) allows for trapping of electrons. Furthermore, due to the dot’s oblong shape, the lateral motion of trapped electrons is primarily in the $y$-direction (see Fig. 1d), such that it couples to the transverse microwave field of the resonator.

To load the dot we use the trap electrode (Fig. 1c, green) to attract reservoir electrons towards the dot, and the resonator guard (blue) to create a barrier between the dot and reservoir. Only if the trap voltage is sufficiently positive, and the resonator guard is sufficiently negative can electrons be loaded and contained in the dot, respectively. When monitoring the resonance frequency shift $\Delta {f}_{0}$ in response to these two voltages, we only see significant signal in an area that is marked by two converging dashed lines in Fig. 2b. The dashed lines are obtained from simulation of the electrostatic potential near the dot (see Methods), and indicate the presence of a barrier between reservoir electrons and electrons in the dot. Well within the predicted trapping region, we observe resonance frequency shifts that depend sensitively on ${V}_{{\rm{trap}}}$ and ${V}_{{\rm{rg}}}$, indicating that trapped electrons in the dot interact with the resonator. The observed shift depends on the number of trapped electrons, which increases for a larger trap voltage, as well as the shape of the electrostatic potential.

### Preparation of small electron clusters

**Fig. 3:**
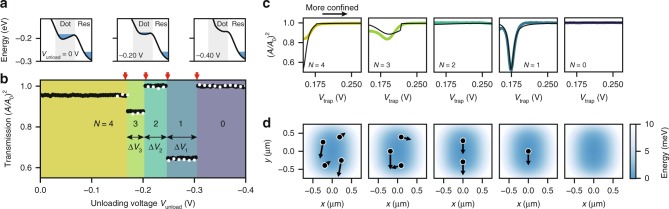
Resonator signatures of few-electron clusters. **a** Schematic of the unloading procedure. At the unloading voltage, the dot’s trap depth decreases for more negative ${V}_{{\rm{unload}}}$. No electrons can occupy the dot at ${V}_{{\rm{unload}}}=-0.4$ V. **b** With decreasing ${V}_{{\rm{unload}}}$, sudden changes in the resonator transmission (black circles, measured at ${V}_{{\rm{trap}}}=0.175$ V and ${V}_{{\rm{tg}}}=0$ V and averaged 500 times) indicate that electrons leave the dot. We observe five distinct plateaus that are reproduced after reloading the dot 8 h later (white circles), and are associated with a constant number of trapped electrons $N$. Red arrows indicate predicted escape voltages for $N=4$ to 1 electrons (left to right) from a single-parameter model, see Methods and Supplementary Table I. **c** Resonator transmission with one to four electrons in the dot, measured by varying the trap curvature using ${V}_{{\rm{trap}}}$. Below ${V}_{{\rm{trap}}}=0.15$ V electron trapping is unstable. The solid black lines are simulated cavity responses (see Methods) and agree qualitatively with the measured resonator transmission spectra. The discontinuity in the simulation for $N=3$ is due to a sudden change in position of the electrons, and is not expected to be visible in the averaged data. **d** Simulated electron configurations in the approximated electrostatic potential, shown for ${V}_{{\rm{trap}}}=0.175$ V. The arrows show the electron motion for the eigenmode that is most strongly coupled to the resonator. The microwave electric field is in the $y$ direction.

To deterministically populate the dot with $N$ electrons, we partially unload the dot using the trap guard electrode (orange in Fig. 1c). A partial unload consists of briefly sweeping the trap guard voltage to ${V}_{{\rm{unload}}} \, < \, 0$, which decreases the trap depth (see Fig. 3a), followed by a measurement of the resonator transmission at $({V}_{{\rm{trap}}},{V}_{{\rm{tg}}})=(0.175,0.0)$ V. The plateaus in resonator transmission shown in Fig. 3b are reproduced after reloading the dot, but are absent when the dot is initially empty. Therefore, each plateau is associated with a constant number of trapped electrons, and the final change in transmission at ${V}_{{\rm{unload}}}=-0.305$ V leaves the dot empty.

The sudden changes in transmission are consistent with single electrons leaving the dot. We show this by modeling the trap as an axially symmetric harmonic well in which the electron configurations can be calculated analytically^25,26^. From the voltage at which the last electron escapes, we estimate unloading voltages for two, three and four electrons, using the effective trap curvature as the only free parameter (see Methods). Red arrows in Fig. 3b indicate these estimates, and agree within 3 mV with the plateau edges. This unloading method therefore allows us to deterministically populate the dot with one to four electrons.

The increasing length of transmission plateaus $\Delta {V}_{N}$ with decreasing $N$ is a sign of strong electron interactions^30,31^, which originate from an unscreened interaction potential on liquid helium^21^. The ratio of interaction energy $U$ to kinetic energy ${k}_{{\rm{B}}}T$, as well as confinement strength $\bar{n}$ quantify electron interaction strength and wavefunction overlap, and predict the formation of Wigner molecules for $\Gamma \, > \,{\Gamma }_{{\rm{c}}}\,=\,U/{k}_{{\rm{B}}}{T}_{{\rm{c}}}\,\approx\,137$ and $n\, < \,{\bar{n}}_{{\rm{c}}}\approx 1/\sqrt{37}$^21,32,33^. Since our experiment operates in the low-temperature ($\Gamma /{\Gamma }_{{\rm{c}}}\approx 9$), low-confinement regime ($\bar{n}/{\bar{n}}_{{\rm{c}}} \approx 0.1$), one would expect electrons in our dot to form Wigner molecules. However, additional measurements, such as a measurement of the melting transition^32,33^, are necessary to exclude an electron-fluid-like state.

While electrons are trapped in the dot, we vary the curvature of the electrostatic potential to gain insight in the electron configurations and electron orbital frequencies. For this measurement, electrons can be trapped and studied for hours, since the trap depth (~10 meV) is large compared with the zero-point energy and thermal energy ($\ll$1 meV). Figure 3c shows five different spectroscopy traces, each corresponding to the different-sized electron clusters from Fig. 3b. To retrieve electron configurations and orbital frequencies, we numerically minimize the total energy of the ensemble and solve the coupled equations of motion^29^. The electron configurations (Fig. 3d) change significantly as electrons are added or removed from the dot, and show correlated electron motion, originating from strong electron interactions. The largest signal in Fig. 3c occurs for a single electron at ${V}_{{\rm{trap}}}=0.175$ V when its orbital frequency is resonant with the resonator. In our model, the orbital frequency of larger clusters remains detuned for all ${V}_{{\rm{trap}}}$ (Supplementary Note 3), which is due to a strong anharmonic component in the electrostatic potential. From the quartic term in this potential, we estimate a single-electron anharmonicity of 85 MHz, which holds promise for creating an electron-on-helium orbital state qubit.

### Single electron properties

**Fig. 4:**
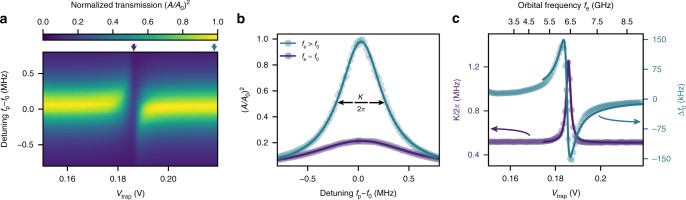
Single electron resonator spectroscopy. **a** Normalized transmission amplitude as function of trap voltage and microwave probe detuning ${f}_{{\rm{p}}}\,-\,{f}_{0}$. **b** Resonator spectra for two values of ${V}_{{\rm{trap}}}$, indicated by arrows on the horizontal axis in **a**. For ${V}_{{\rm{trap}}}$ = 0.184 V (purple circles) the electron is resonant with the cavity, whereas for ${V}_{{\rm{trap}}}\,=\,0.23$ V the electron is far off-resonant (turquoise circles). The resonant trace illustrates the sensitivity of our device to a single electron. **c** Cavity resonance frequency shift (turquoise circles, right axis) and resonator decay rate (purple circles, left axis) obtained by fitting the Lorentzian resonator spectra from **a**. The solid turquoise line is a fit to a model that yields a coupling strength near resonance of $g/2\pi\,=\,4.8\,\pm\,0.3$ MHz and total electron linewidth $\gamma /2\pi =77\pm 19$ MHz. The top horizontal axis displays how the electron orbital frequency varies as function of ${V}_{{\rm{trap}}}$, and shows a crossing with the resonator (${f}_{{\rm{e}}}=6.4$ GHz) at ${V}_{{\rm{trap}}}\,=\,0.184$ V.

We now focus on a single trapped electron and investigate its properties by tuning the orbital frequency into resonance with the resonator. Figure 4a shows a crossing of the orbital frequency with the resonator around ${V}_{{\rm{trap}}}=0.184$ V, which is accompanied by a rapid change in $\Delta {f}_{0}$ (Fig. 4c). By fitting the measured frequency shift to a model, which takes into account one orbital mode coupled to a single resonator mode^34^, we obtain a single-electron-photon coupling strength $g\,=\,2\pi\,\times\,(4.8\,\pm\,0.3)$ MHz and electron linewidth $\gamma ={\gamma }_{1}/2+{\gamma }_{\varphi }=2\pi \times (77\pm 19)$ MHz. The coupling strength is large compared with the resonator linewidth ($\kappa /2\pi \approx 0.5$ MHz), indicating that each photon measures the presence of the electron, and the coupling is similar to that measured in semiconducting quantum dot cQED architectures^13^. In addition, our estimate of the anharmonicity (Supplementary Fig. 7) is similar to that in superconducting qubits, indicating that with a reduced linewidth the orbital state of a single electron on helium can be used as a qubit.

The total linewidth $\gamma$ is three orders of magnitude larger than expected from the electron-phonon coupling in ^4^He and charge noise from the bias electrodes, respectively ($\gamma /2\pi \, < \,0.1$ MHz)^2^. We identify the dominant source of excess noise as classical helium fluctuations in the dot, caused by the pulse tube refrigerator (Supplementary Note 6). This is corroborated by a measurement of the crossing voltage as function of time, which shows spectral features of the pulse tube refrigerator (see Supplementary Fig. 12). To estimate the dephasing rate due to helium fluctuations, we estimate an electron’s sensitivity to helium fluctuations from electrostatic simulations ($\partial {f}_{{\rm{e}}}/\partial {t}_{{\rm{He}}}\,\approx\,80$ MHz nm^−1^) and independently measure helium fluctuations ($\Delta {t}_{{\rm{He}}}\approx 1.4$ nm), yielding ${\gamma }_{\varphi }/2\pi \approx 110$ MHz. Therefore, we expect the single electron linewidth to be limited by dephasing due to helium level fluctuations.

## Discussion

Reducing the linewidth and increasing the coupling strength offers a path toward the strong coupling regime, which has recently been achieved for the cyclotron motion of large electron ensembles on liquid helium^35^. In the strong coupling regime, direct measurement of the electron orbital frequencies using two-tone spectroscopy^36^ may bring to light new microwave features of strongly correlated electron states^37^. Since the orbital frequencies span tens of GHz (see Supplementary Fig. 6) this measurement would benefit from a frequency tunable microwave resonator^14^, a feature that can be embedded in a future device.

To reach the strong coupling regime with future electron-on-helium dots, one can passively or actively reduce the vibrations that excite the helium surface^38,39^ and engineer a dot geometry that has a reduced sensitivity to classical helium vibrations. Preliminary simulations of a dot with a less sensitive electrode geometry show a hundredfold reduction in linewidth. In addition, a microwave resonator made of a high kinetic inductance superconductor can enhance the coupling strength by more than three times via an increased characteristic impedance^10,40^. The combination of reduced sensitivity and increased coupling strength would put a single electron on helium in the strong coupling regime.

In conclusion, we have integrated an electron-on-helium dot with a superconducting microwave resonator and observed distinct resonator signatures of small electron clusters consisting of up to four electrons. The large anharmonicity and coupling strength of a single electron on helium hold promise for creating an electron-on-helium qubit, which can be readily integrated with superconducting qubits while leveraging established protocols. Finally, when combined with a magnetic field gradient, the orbital state offers a clear path towards control of a single electron spin.

## Methods

### Fabrication

First an 80 nm thick Nb ground plane was evaporated onto a high-resistivity ($> 10$ k$\Omega$ cm) Si $\langle 100\rangle$ wafer, followed by deposition of a 100 nm thick silicon oxide sacrificial layer, which was used to protect the Nb ground plane during the following etch steps. The micro-channels were defined using a Raith EBPG-5000+ electron beam lithography system and etched using a CHF${}_{3}$/SF${}_{6}$ chemistry, immediately followed by an HBr/O_2_ etch. In the second step the resonator center pins were defined using e-beam lithography. After development, evaporation of a 150 nm thick Nb layer and lift-off, the center pins remained on the bottom of the micro-channel. To improve robustness of the device and avoid electrical breakdown at low temperatures, we etched away an additional $\sim$400 nm of Si substrate in between the resonator center pins. To this end, another layer of 80 nm thick silicon oxide was deposited, after which the additional Si was etched with the previously described etch chemistry. The silicon oxide layer was removed using buffered HF and a deionized water rinse.

### Measurements

All measurements were performed in an Oxford Triton 200 dilution refrigerator with a base temperature of 25 mK. The chip was mounted in a custom-designed hermetic sample cell and sealed with indium to prevent superfluid helium leaks. Helium was supplied to the sample cell from a high purity ^4^He gas cylinder and, using a control volume ($V\approx 25$ cm^3^) in a gas handling system, we were able to introduce a controlled amount of helium to the sample cell. The experiment was performed in a regime where the channel was almost full and the liquid helium film was stabilized due to surface tension^41^.

Electrons were captured on the helium surface by thermal emission from a tungsten filament situated above the chip, while applying a positive voltage to the resonator DC bias electrode (${V}_{{\rm{res}}}=3.0$ V) and a negative bias voltage to the filament. We assume electrons in the reservoir were distributed uniformly across the resonator and estimate the electron density from the resonator voltage at which electrons can no longer be contained on the resonator, as depicted by the sudden increase in $\Delta {f}_{0}$ in Fig. 2a. At ${V}_{{\rm{res}}}^{{\rm{th}}}=0.18$ V, electrons flow onto the Nb ground plane and we estimate the electron density

1 $$n\approx \frac{{\varepsilon }_{0}{\varepsilon }_{{\rm{He}}}}{e{t}_{{\rm{He}}}}{V}_{{\rm{res}}}^{{\rm{th}}}=9\times 1{0}^{12}\ {{\rm{m}}}^{-2},$$

where ${t}_{{\rm{He}}}$ is the helium thickness, ${\varepsilon }_{{\rm{He}}}=1.056$ is the dielectric constant of helium and $e$ is the elementary charge. This density corresponds to ~$1{0}^{5}$ reservoir electrons, whose orbital frequency stayed far detuned from ${f}_{0}$ during experiments with electrons in the dot.

The pulse tube refrigerator is a continuous source of mechanical vibrations which excites the liquid helium surface. These vibrations were detected by the microwave resonator as a slowly varying resonance frequency jitter, with a standard deviation of ~$6.8$ kHz in the absence of reservoir electrons (Supplementary Fig. 5). This jitter complicated the measurement of small resonance frequency shifts due to trapped electrons, which were typically of the same order as the jitter. However, since the dominant frequency components in the mechanical noise spectrum were below 10 Hz, we circumvented this issue by sweeping electrode voltages faster than 1/10 Hz^−1^, such that signatures of trapped electrons became visible after averaging.

### Electrostatic simulations of the dot

The electrostatic potential near the dot was obtained by solving Poisson’s equation using the finite element method with ANSYS MAXWELL. We separately solve the potential for each electrode that contributes to the dot potential by applying 1 V on a single electrode while keeping all other electrodes grounded. We minimize numerical noise in the potential by increasing the vertex density in the center of the dot and imposing strong convergence criteria. For post-processing the potential values are cast to a regular Cartesian grid using interpolation.

The two converging dashed line segments in Fig. 2b are obtained by considering both the potential along the channel and the reservoir density. The reservoir density $n$ sets the chemical potential of the reservoir via ~${e}^{2}n{t}_{{\rm{He}}}/{\varepsilon }_{0}{\varepsilon }_{{\rm{He}}}$, and for larger $n$, ${V}_{{\rm{rg}}}$ must be more negative to maintain a barrier between reservoir and dot (Fig. 2d). For our device, this non-zero barrier condition is captured by a line segment with slope 1.15. The reservoir density $n$ determines the offset of this line segment, and was measured by increasing ${V}_{{\rm{trap}}}$ until electron transport occurred onto the trap electrode. From an equation similar to Eq. (1) we find $n\approx 4\times 1{0}^{12}$ m${}^{-2}$. The horizontal line segment was found by finding the minimum ${V}_{{\rm{trap}}}$ for which the reservoir extends left of $x=1.5$ µm at ${V}_{{\rm{rg}}}=0$. Figure 2c shows a situation above this threshold, for which the loading operation should result in trapped electrons.

### Unloading the dot

The dot was unloaded by sweeping the trap guard to ${V}_{{\rm{tg}}}={V}_{{\rm{unload}}} \, < \, 0$ while keeping all other electrodes constant at $({V}_{{\rm{res}}},{V}_{{\rm{trap}}},{V}_{{\rm{rg}}})=(0.6,0.15,-0.4)$ V. The electrodes were then ramped back to $({V}_{{\rm{trap}}},{V}_{{\rm{tg}}})=(0.175,0)$ V in order to probe the resonator transmission. A single unloading procedure took about 10 ms, which is limited by the corner frequency of the trap guard electrode RC-filter. The ramp speed did not change the charging diagram of Fig. 3b.

To confirm that changes between transmission plateaus in Fig. 3b are associated with single electron transport, we simulated unloading using a combination of electrostatic simulations and analytical calculations. Even though the electrode geometry in the dot produced a complex and anharmonic trapping potential on the scale of the dot (8 × 4 µm), the small extent of the electron ensemble (0.5 × 0.5 µm) allowed us to simulate the unloading with an axially symmetric harmonic well. The unloading voltage ${V}_{{\rm{unload}}}$ decreased the trap depth and resulted in unloading of the dot. We modeled this process as a linear decrease in barrier height: ${V}_{b}={V}_{{\rm{bar}}}+\beta {V}_{{\rm{unload}}}$, where ${V}_{{\rm{bar}}}=22$ meV was obtained from electrostatic simulations and $\beta$ was determined from the final jump ${(A/{A}_{0})}^{2}$ in Fig. 3b. The energies of the clusters were calculated analytically^27^, which resulted in the unloading voltages ${V}_{{\rm{unload}}}^{(N)}$:

2 $${V}_{{\rm{unload}}}^{(1)}=-\frac{{V}_{{\rm{bar}}}}{\beta }=-0.305\ {\rm{V}}$$

3 $${V}_{{\rm{unload}}}^{(2)}={V}_{{\rm{unload}}}^{(1)}+\frac{3}{4}\frac{{E}_{0}}{\beta e}$$

4 $${V}_{{\rm{unload}}}^{(3)}={V}_{{\rm{unload}}}^{(1)}+1.31037\frac{{E}_{0}}{\beta e}$$

5 $${V}_{{\rm{unload}}}^{(4)}={V}_{{\rm{unload}}}^{(1)}+1.83545\frac{{E}_{0}}{\beta e}$$

where

6 $${E}_{0}={\left(\frac{{m}_{{\rm{e}}}{\omega }_{{\rm{e}}}^{2}{e}^{4}}{2{\left(4\pi \right)}^{2}{\varepsilon }_{0}^{2}{\varepsilon }_{{\rm{He}}}^{2}}\right)}^{\frac{1}{3}}$$

and depends only on the trap curvature at the unloading point (${\omega }_{{\rm{e}}}$), electron mass (${m}_{{\rm{e}}}$) and other physical constants. Best agreement between model and experiment was found with an effective trap curvature ${\omega }_{{\rm{e}}}/2\pi \,=\,26$ GHz, which produces the red arrows in Fig. 3b.

If the dot had initially contained five electrons, our model would have predicted an additional plateau starting at ${V}_{{\rm{unload}}}^{(5)}$ = −0.127 V. Since we did not observe this plateau we concluded the trap was initially loaded with $N=4$ electrons.

### Modeling of resonator transmission spectra

To accurately model the resonator transmission spectra with electrons in the dot, we needed a more sophisticated model of the electrostatic potential than an axially symmetric harmonic well. Therefore, the electrostatic potential was approximated by

7 $$E/e={\alpha }_{0}({V}_{{\rm{trap}}}){x}^{2}+{\alpha }_{1}({V}_{{\rm{trap}}}){y}^{2}+{\alpha }_{2}({V}_{{\rm{trap}}}){y}^{4}.$$

Without a quartic term, the method described below predicts crossings for all electron clusters at equal ${V}_{{\rm{trap}}}$, which is inconsistent with experiment. Eq. (7) represents a minimal model that reproduces the observed resonator transmission spectra. The coefficients ${\alpha }_{i}$ were obtained by first fitting Eq. (7) to the electrostatic potential obtained via finite element modeling, and were then slightly adjusted to reproduce the experimental traces, using the following method.

For a particular trap voltage the electron configurations were found through numerical minimization of the total energy, which included a small screening correction to the interaction energy due to the metal electrodes under the electrons. In addition, we neglected the kinetic term in the total energy, since at $T=25$ mK the kinetic energy is approximately three orders of magnitude smaller than the interaction energy. Next, using the electron positions as input, the cavity frequency shift and orbital frequencies were determined by solving the linearized equations of motion of the coupled cavity-electron system. We then took the strongest-coupled orbital frequency ${\omega }_{e}$ and calculated its effect on the resonator via

8 $$\frac{A}{{A}_{0}}=\left|\frac{\sqrt{{\kappa }_{1}{\kappa }_{2}}}{i({\kappa }_{1}\,+\,{\kappa }_{2}\,+\,{\kappa }_{{\rm{int}}})/2\,-\,\chi ({\omega }_{0})}\right|,$$

where ${\kappa }_{1,2,{\rm{int}}}$ represents the coupling through port 1 and 2 of the resonator and the internal loss rate, respectively. In addition, the susceptibility is given by

9 $$\chi ({\omega }_{0})\,=\,\frac{{g}^{2}}{({\omega }_{0}\,-\,{\omega }_{{\rm{e}}})\,+\,i\gamma }.$$

$g/2\pi$ was fixed at 5 MHz (estimated from the resonator geometry, see Supplementary Note 1) and $\gamma /2\pi$ was adjusted to get good agreement for $N=1$. $\gamma$ was not further adjusted for $N \, > \, 1$, since for those electron clusters all orbital modes stayed far detuned and the modeled traces only weakly depended on $\gamma$. With this method we obtained the resonator responses shown as solid black traces in Fig. 3c.

We obtained better agreement between the data and model for one and two electrons, compared with three and four electrons. This can be attributed to the larger size of the three and four-electron clusters, since the approximation of the electrostatic potential in Eq. (7) only holds for small $x,y$. In addition, each resonator transmission spectrum was averaged 500 times which blurs sharp features, such as the one in the modeled three-electron trace.

The anharmonicity of a single electron was estimated by treating the ${y}^{4}$ term in Eq. (7) as a perturbation to the harmonic oscillator Hamiltonian. We define the anharmonicity $\alpha$ as $\hslash \alpha =({E}_{2}-{E}_{1})-({E}_{1}-{E}_{0})$, where ${E}_{{\rm{n}}}$ are the perturbed eigenenergies. Near the crossing with the resonator we find ${\alpha }_{2}\approx 0.014$ µm^−4^, leading to

10 $$\frac{\alpha }{2\pi }=\frac{1}{2\pi }\frac{3e{\alpha }_{2}\hslash }{{m}_{{\rm{e}}}^{2}{\omega }_{{\rm{e}}}^{2}}\approx 85\ {\rm{MHz}}.$$

### Extracting single electron properties

To extract $g$ and $\gamma$ from the data in Fig. 4c, we used the same model for the resonator transmission as in Eq. (8), which was based on input-output theory and assumed that one orbital mode coupled to the resonator. To fit the frequency shift vs. trap voltage, we needed to know ${\omega }_{{\rm{e}}}$ as function of ${V}_{{\rm{trap}}}$. We used quadratic fits to a finite element model of the electrostatic potential, which accurately predicted the single-electron crossing voltage, to find the dependence of ${\omega }_{{\rm{e}}}$ on ${V}_{{\rm{trap}}}$. For the data in Fig. 4c, this method predicted a sensitivity near the crossing of $\partial {f}_{{\rm{e}}}/\partial {V}_{{\rm{trap}}}=95$ GHz V^−1^ (see Supplementary Fig. 7) and also gives the top horizontal axis in Fig. 4c.

Since the measured frequency shift remained less than a linewidth, the phase ($\Delta \varphi$) was a direct measure of the cavity frequency shift and the conversion was made via $\Delta \varphi =\arctan \left(\Delta {f}_{0}/{\kappa }_{{\rm{tot}}}\right)\approx \Delta {f}_{0}/{\kappa }_{{\rm{tot}}}$, where ${\kappa }_{{\rm{tot}}}\,=\,{\kappa }_{1}\,+\,{\kappa }_{2}\,+\,{\kappa }_{{\rm{int}}}$. Using the simulated ${\omega }_{{\rm{e}}}$ vs. ${V}_{{\rm{trap}}}$, we fit the measured cavity frequency shift to $\Delta {f}_{0}=\Delta \varphi {\kappa }_{{\rm{tot}}}$, which gave the values listed in the main text. Quoted uncertainties were fit uncertainties.

## Supplementary Information

Supplementary Information

## Data Availability

The data and simulation files that support the findings of this study are available on reasonable request from the authors.
