# Supplementary material for: Coupling a single electron on superfluid helium to a superconducting resonator
Source: Nat Commun. 2019 Nov 22;10:5323. doi: 10.1038/s41467-019-13335-7 (PMC6874564; doi:10.1038/s41467-019-13335-7)
Supplement: Supplementary file 1 — Supplementary Information [file 41467_2019_13335_MOESM1_ESM.pdf]

Supplementary Information

for

Coupling a single electron on superfluid helium to a superconducting resonator

Koolstra *et al.*

Supplementary Note 1. Microwave resonator design and measurements

A. Design of the differential microwave mode

To couple to the orbital electron state we use a superconducting microwave resonator consisting of two center pins surrounded by a ground plane. This geometry is schematically depicted in Supplementary Fig. 1. In general, this resonator geometry supports two types of modes. For the mode of interest, the pins carry an equal but opposite voltage at any point along the cross section. The microwave electric field is approximately constant between the two center pins, such that it can couple efficiently to the lateral motion of a single electron or a single row of electrons in the center of the micro-channel.

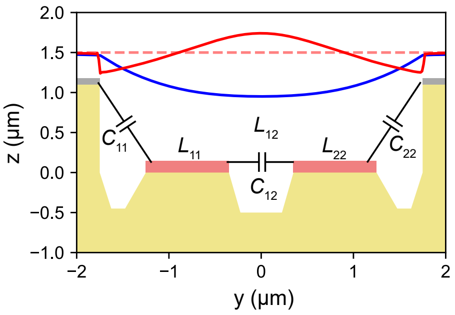

Supplementary Figure 1. Schematic representation of the capacitances C_{ij} and inductances L_{ij} involved in a differential pair with two Nb center pins (red) and a ground plane (gray) on a Si substrate (yellow). The general shape of the DC potential (solid blue line) and microwave electric field E_y (solid red line) are evaluated at the helium filling height $z = 1.2$ μm. For the latter, a dashed line indicates $E_y = 0$.

The essential microwave properties, e.g. the impedance Z and resonance frequency f_0, can be extracted from the capacitances and inductances from Supplementary Fig. 1. The inductances and capacitances can be written in a matrix as follows:

$$\mathcal{L} = \begin{pmatrix} L_{11} & L_{12} \\ L_{21} & L_{22} \end{pmatrix} \quad \text{and} \quad \mathcal{C} = \begin{pmatrix} C_{11} & C_{12} \\ C_{21} & C_{22} \end{pmatrix}. \tag{1}$$

Given our model geometry, each of the entries can be simulated using a finite element simulation package (e.g. Ansys Electronics Desktop). The impedance of the microwave differential mode is given by

$$Z_{\text{diff}} = 2\sqrt{\frac{L_{11} - L_{12}}{C_{11} + |C_{12}|}}. \tag{2}$$

Without kinetic inductance (we estimate a kinetic inductance fraction of only 5%) we estimate the characteristic impedance $Z_{\text{diff}} \approx 90\,\Omega$. Additionally, we find an expression for the expected resonance frequency for the quarter wavelength differential mode:

$$f_0 = \frac{1}{4\ell}\frac{1}{\sqrt{(L_{11} - L_{12})(C_{11} + |C_{12}|)}}, \tag{3}$$

where ℓ is the length of the resonator measured from the tip to the point where the two center pins meet.

B. Electron-photon coupling

The coupling strength of a single electron to a single microwave photon can be estimated from the dipole energy

$$\hbar g = \boldsymbol{d} \cdot \mathbf{E}. \tag{4}$$

where $\boldsymbol{d} = e y_{\text{zpf}} \hat{\mathbf{y}}$ is the dipole moment, e is the electron charge and $y_{\text{zpf}} = \sqrt{\hbar/2m_{\text{e}}\omega_{\text{e}}}$ is the zero point motion of the electron in the $\hat{\mathbf{y}}$-direction. The electric field $\mathbf{E} = E_y \hat{\mathbf{y}}$, where E_{y} is the electric field in the y-direction generated

by the zero-point fluctuations of the microwave resonator $V_{\rm rms}$. The latter quantity can be estimated from the fact that on resonance half of the cavity's zero point energy is stored in the capacitor, such that

$$\frac{1}{4}\hbar\omega_0 = \frac{1}{2}\frac{1}{T}\int_0^T \mathrm{d}t\cos^2(\omega_0 t)\int \mathrm{d}^3 r\varepsilon E^2(r) = \frac{1}{4}CV_{\rm zpf}^2 = \frac{1}{2}CV_{\rm rms}^2. \tag{5}$$

This can be further simplified using the relations $\omega_0 = \sqrt{1/LC}$ and $Z = \sqrt{L/C}$, such that Eq. (5) yields $V_{\rm rms} = \omega_0\sqrt{\hbar Z/2}$. Plugging this in Eq. (4) gives

$$g/2\pi = \boldsymbol{d}\cdot\mathbf{E} = \frac{1}{2}eE_y f_0\sqrt{\frac{Z}{m_{\rm e}\omega_{\rm e}}}. \tag{6}$$

For realistic experimental values of $E_y \approx 2\times10^5$ V/m (see Supplementary Fig. 11b), $Z = 90\ \Omega$ and $f_0 = \omega_0/2\pi = 6.45$ GHz, we arrive at $g/2\pi \approx 5.0$ MHz.

C. On-chip filter and microwave resonator characterization

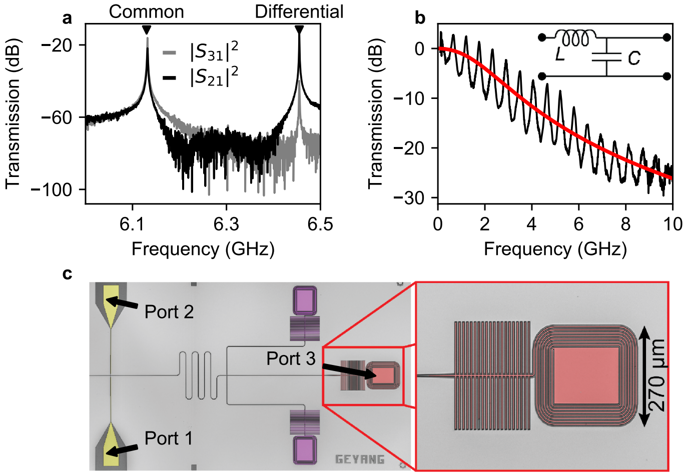

Supplementary Figure 2. Characterization of the on-chip LC-filter and the effect of the DC-bias line (a) Comparison between the microwave transmission through the DC bias port ($|S_{31}|^2$) and the resonator's transmission $|S_{21}|^2$. The ports 1, 2 and 3 are defined in the optical micrograph in (c). (b) Microwave transmission of the on-chip LC-filters can be modeled using a two port circuit model (solid red line) as shown in the inset. (c) Optical micrograph of the spiral inductor that is part of the device's on-chip LC-filters. During the experiment these pads are flooded with helium and have to be positively biased, such that they accumulate unwanted electrons.

To characterize the resonator we measure its transmission by driving and detecting through the yellow microwave feed lines in Supplementary Fig. 2c. The resulting transmission $|S_{21}|^2$ shows two peaks, separated by 300 MHz, which we identify as the common and differential mode of the microwave resonator. We identify the differential mode by also detecting the microwave transmission through the resonator DC bias line. Ideally, the transmission through the DC bias line is fully suppressed for the differential mode. However, due to asymmetry in the microwave field due to fabrication imperfections $|S_{31}|^2$ only shows a 27 dB reduced peak amplitude at 6.45 GHz. This is in contrast to the lower resonance at 6.15 GHz, for which the transmission amplitude increases. This indicates that the differential (common) mode has a resonance frequency of 6.45 (6.15) GHz.

Without electrons or helium on top of the resonator, S_{ij} is accurately described by

$$S_{ij} = \frac{\sqrt{\kappa_i\kappa_j}}{(\omega-\omega_0)+i\kappa_{\rm tot}/2}, \tag{7}$$

where $\kappa_{\rm tot} = \kappa_1+\kappa_2+\kappa_3+\kappa_{\rm int}$ is the total line width and κ_{1-3} are the coupling rates through ports 1-3. From a fit to this model, and defining the loaded quality factor as $Q_L = \omega_0/\kappa_{\rm tot}$, we find $Q_L \approx 18\times10^3$ for the differential

mode. Additionally, Eq. (7) allows us to estimate κ_3, since if ports 1 and 2 have equal coupling ($\kappa_1 = \kappa_2 = \kappa_c$), then

$$\frac{\kappa_3}{\kappa_c} = \frac{|S_{31}(\omega = \omega_0)|^2}{|S_{21}(\omega = \omega_0)|^2}. \tag{8}$$

Therefore, the 27 dB reduced transmission amplitude indicates that $\kappa_3/\kappa_c = 2 \times 10^{-3}$ and we conclude that Q_L is not limited by leakage through the DC bias port.

We also measure the transmission of the on-chip microwave filters, using a separately fabricated chip containing just the filters and a through line for calibration of the amplitude. The result of this measurement is shown in Supplementary Fig. 2b. The response shows a standing wave pattern, most likely due to an impedance mismatch between the chip and the printed circuit board. Apart from the oscillations, the overall response can be modeled well by a two-port LC-circuit as shown in the inset. We find that a capacitance of 4 pF and an inductance of 2.5 nH describe the response well (as shown by the red line). At the differential mode resonance frequency, the reflection is found to be 18 dB.

D. Experimental setup

Supplementary Fig. 3 shows a schematic diagram of the setup, not including the gas handling system that supplies helium to the sample box. These details can be found in the supplement of Ref. [1].

1. *Microwave setup*

All microwave measurements were done with a Keysight PNA-X Network Analyzer. The transmitted signal from the microwave resonator is amplified by a Josephson parametric amplifier which provides a gain of approximately 20 dB at the cavity resonance frequency. The signal is subsequently amplified by a high electron mobility amplifier at 4 K (Low Noise Factory LNF-LNC48C, gain 38 dB) and a room temperature amplifier (Miteq AFS3-00101200, gain 28 dB). In addition, DC blocks (Inmet 8039) inserted in the in and output lines prevent ground loops.

2. *DC filtering*

Each DC electrode is low-pass filtered using a three stage filter attached to the mixing chamber plate of the refrigerator. The first two stages are combined on a custom designed printed circuit board, which is situated in a copper enclosure filled with Eccosorb CR117. The PCB contains pairs of long meandering traces [2] to increase the effective contact length with the lossy ferrite, and R-C filters with cut-off frequencies in the range 2-200 Hz. The third stage consists of a Minicircuits ZX75LP-30+ low pass filter that attenuates noise in the 30-3000 MHz range.

Additionally, an on-chip LC filter ($L \approx 2.5$ nH, $C \approx 4$ pF) for each electrode (attenuation of 18 dB at 6.5 GHz) further reduces the number of high frequency thermal photons that would otherwise degrade the cavity quality factor or adversely affect the electron motional state. [3]

E. Resonator response to superfluid helium

As liquid helium fills the cylindrical reservoir below the chip, capillary action causes the channels to fill with liquid helium. As helium is added to the reservoir, the distance of the helium to the chip h decreases and the micro-channel fills according to Jurin's law:

$$\rho g h = \frac{\sigma}{R}, \tag{9}$$

where $\rho = 145$ kg m^{-3} is the density of liquid helium, $\sigma = 3.78 \times 10^{-4}$ N m^{-2} is the surface tension and R is the radius of curvature of the helium-vacuum interface. To first order this equation states that the surface of the helium assumes the shape of a quadratic form $z(x, h)$:

$$z(x, h) = d_0 + \frac{\rho g h}{2\sigma}\left(x^2 - \frac{w^2}{4}\right), \tag{10}$$

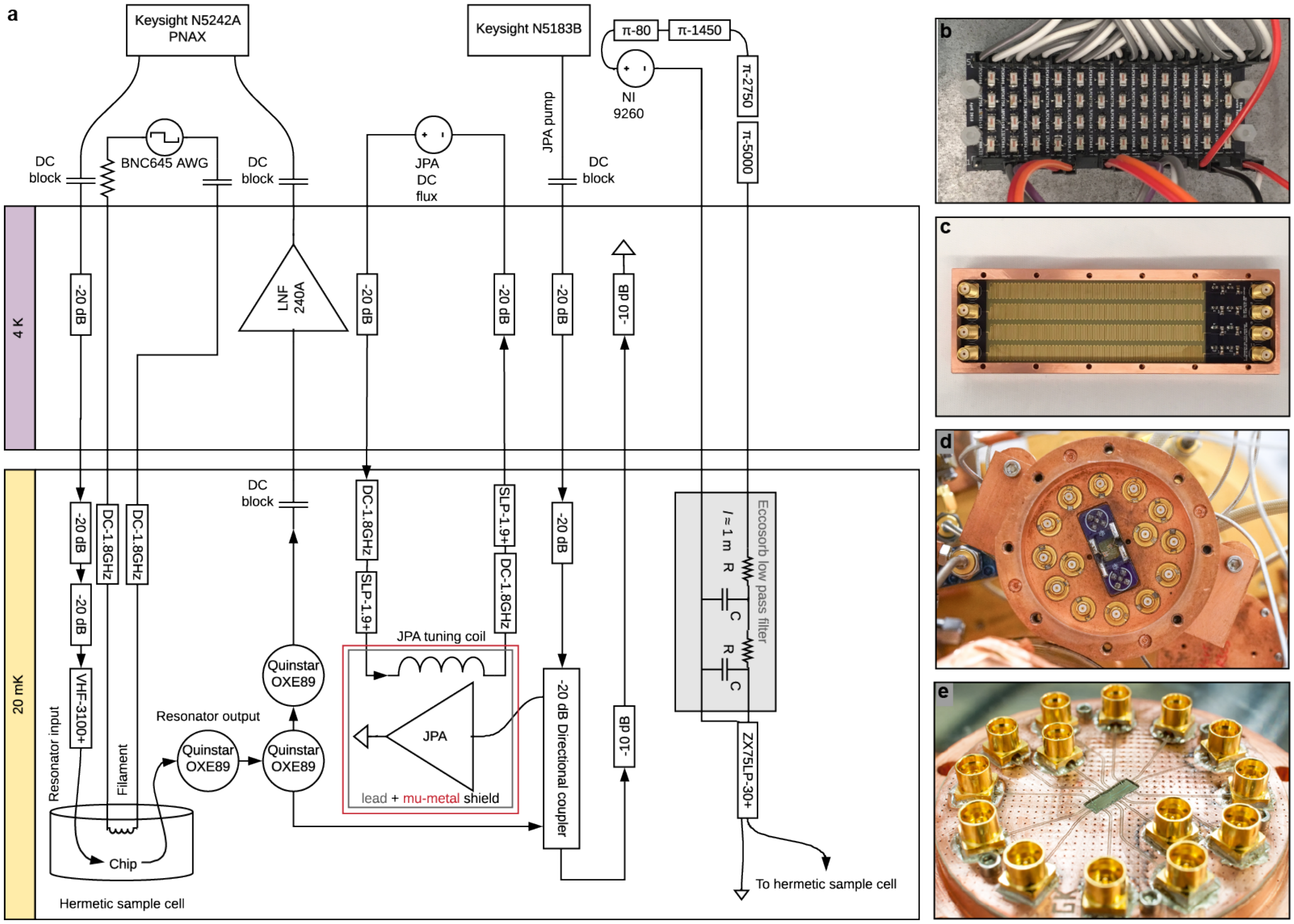

Supplementary Figure 3. Schematic and details of the experimental setup (a) The colored boxes represent different stages of the dilution refrigerator (50K, still and 100 mK plate not shown). Photographs of setup details are shown on the right. (b) Minicircuits π-filters (labeled as π-80, π-1450, π-2750 and π-5000 in (a)) mounted on a PCB at room temperature protect each pair of DC wires before entering the cryostat (c) copper enclosure containing RC filters and meandering wire (Eccosorb not shown) (d) Photograph of the inside of the sample box lid, showing 14 + 2 hermetic SMP connections which carry DC and microwave connections into the sample cell. The PCB in the center contains two tungsten filaments which are used as an electron source. (e) PCB with chip mounted to the bottom of sample cell. This part attaches to the lid shown in (d) and is sealed using a 0.020" OD indium seal applied around the circumference of the pedestal.

where $d_0 = 1.2$ µm and $w = 3.5$ µm are the depth and width of the channel, respectively. The level of the liquid in the center of the channel is

$$z(0,h) = t_{\mathrm{He}} = \max\left(0, d_0 - \frac{\rho g h}{2\sigma}\frac{w^2}{4}\right). \tag{11}$$

The resonator frequency shift due to helium is depicted in Supplementary Fig. 4, where four different regions can be identified. In region I a $\sim$30 nm Vanderwaals film covers the entire sample, resulting in only a small frequency shift. Region II is characterized by a large jump in $\Delta\omega_0$ followed by a plateau. In this region helium fills the channel due to capillary action, until $h = 0$. The plateau in $\omega_0/2\pi$ from 30-110 puffs can be explained by the channel geometry and the maximum value of h set by helium reservoir depth. From Eq. (11), we estimate the helium depth in the plateau to vary from 1.0 to 1.2 µm. Introducing more helium results in filling the entire upper half plane (region III). The resonance frequency shift increases until the helium has filled the mode volume of the resonator. Beyond this point the electric field is negligible and, therefore, adding more helium does not result in an extra frequency shift (region IV).

At each point along the curve of Supplementary Fig. 4a, we repeatedly measure the resonance frequency ω_0. The spread in ω_0 at a particular helium filling is a result of superfluid helium vibrations that originate from continuous

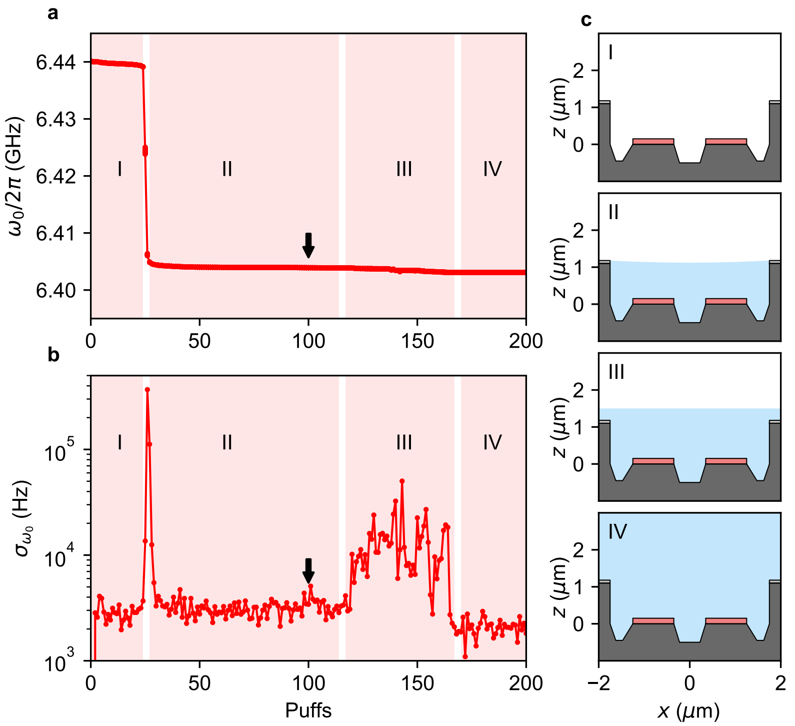

Supplementary Figure 4. Resonator response to adding liquid helium to the sample cell (a) Resonance frequency shift $\Delta\omega_0/2\pi$ (red dots) and (b) Resonance frequency jitter σ_{ω_0} (red dots) as function of the number of ^{4}He gas puffs introduced to the sample cell. One puff corresponds to approximately 25 cc of ^{4}He gas at STP. The experiment is performed with 100 puffs in the sample cell (black arrow). (c) Schematics of the channel showing the helium level (light blue) in each of the four regions indicated as red bands in (a) and (b).

excitation from the pulse tube, and building vibrations that couple into the cryostat through its frame. In Supplementary Fig. 4b we plot the standard deviation σ_{ω_0} of 25 measurements of ω_0. Note that each measurement of ω_0 was acquired faster than the dominant frequency in the helium vibrational spectrum, such that the peak was not artificially broadened. Therefore, σ_{ω_0} gives a direct indication of the helium vibrations on the resonator.

Supplementary Fig 4b shows that helium vibrations are worst at the transition from region I to region II, i.e. just before the channel fills up with helium. In region II, the capillary action stabilizes the helium film and suppresses vibrations. An additional increase in helium vibrations is seen when the channel is completely full and capillary action no longer stabilizes the film.

Since helium vibrations are detrimental to the coherence of the electron orbital state, we decide to work at a point where σ_{ω_0} is at a minimum. The black arrow in Supplementary Fig. 4b shows this point. To further quantify the helium vibrations at this point, we monitor the resonance frequency as function of time and observe periodic oscillations with dominant frequencies less than 10 Hz (Supplementary Fig. 5a,b). From the helium-resonator coupling (5 kHz nm^{-1}) we estimate the magnitude of classical helium fluctuations to be $\Delta t_{\mathrm{He}} = 1.4$ nm. The resonator frequency fluctuations due to these vibrations increases by a factor of five when reservoir electrons are present (Supplementary Fig. 5c) because electrons couple more strongly to the resonator than helium.

Even though the magnitude of the jitter is less than a resonator linewidth κ_{tot}, the variation of the resonance frequency shift over time obscures small frequency shifts due to electrons near the dot. We have tried various ways to minimize this noise, including turning off the pulse tube and working at elevated temperature to reduce the quality factor of the surface vibrations. Unfortunately, we see no improvement with the pulse tube turned off until $T > 0.3$ K and working at these temperatures introduces thermal noise which degrades the electron motional state noticably (see Supplementary Note 5). To circumvent the issue of the resonator jitter, we sweep the trap or guard voltages at a rate much faster than the dominant helium vibration frequency, such that frequency shifts from electrons in the dot become quickly apparent after averaging. A more quantitative description of the effect of helium vibrations on trapped electrons is given in Supplementary Note 6 A.

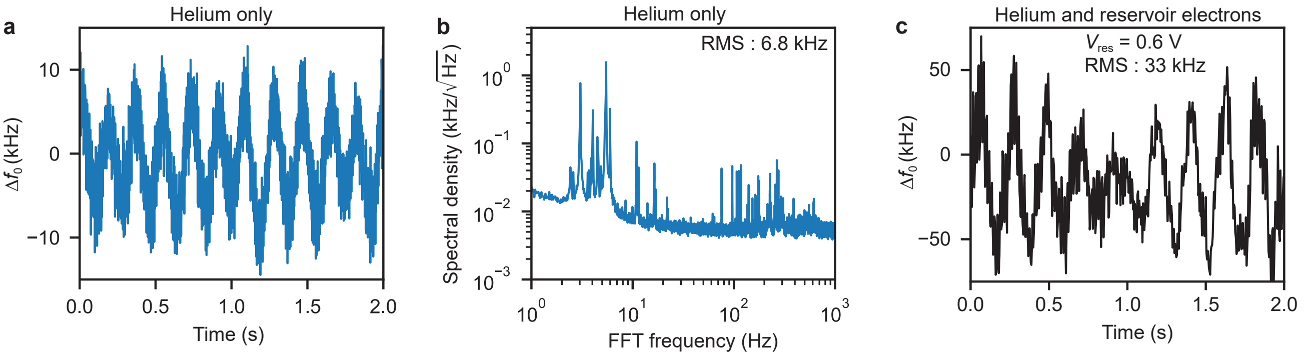

Supplementary Figure 5. Using the microwave resonator to detect liquid helium vibrations (a) Resonance frequency jitter due to helium vibrations measured at $T = 25$ mK without reservoir electrons. (b) Most of the spectral density for the data in (a) lies below 10 Hz. Nearly all of the resonance frequencies in this region can be associated with a multiple of 1.4 Hz, the frequency of the pulse tube refrigerator. The quality factor of these modes are at least a few hundred. (c) After depositing reservoir electrons the frequency jitter increases. Note the difference in scale compared with (a). All time traces are taken with a microwave tone on resonance and converting the phase fluctuations to resonance frequency fluctuations using the resonator linewidth κ_{tot}.

F. Estimate of liquid helium ringdown time

It may seem surprising that turning off the pulse tube refrigerator has little effect on the coherence of a single electron. However, at $T = 20$ mK liquid helium is a very low-viscosity superfluid whose excitations have been exploited in several optomechanics experiments [4–6]. We estimate the damping time for liquid helium surface waves below.

The damping time of the reservoir fluctuations can be estimated from $\tau = Q/\omega_{\mathrm{He}}$, where Q is the quality factor of the helium surface vibrations and ω_{He} the fundamental vibration mode of the helium surface. We may treat the helium surface in the cylindrical reservoir as a drum with clamped edges, and the fundamental vibration frequency is related to the wavelength via the anomalous dispersion $\omega_{\mathrm{He}}^2 = g k_{\mathrm{He}}$, where we neglect the short-wavelength contribution due to surface tension. Clamped boundary conditions imply $k_{\mathrm{He}} = p_{01}/r$, where $p_{01} \approx 2.4$ is the first zero of the Bessel function J_0. This gives a fundamental frequency $f_0 = \frac{1}{2\pi}\sqrt{g p_{01}/r} \approx 15$ Hz, assuming a cylindrical reservoir of diameter 5 mm. The dominant mode in the spectrum of Supplementary Fig. 5b occurs at 5.6 Hz. This discrepancy may be due to larger ($r \approx 15$ mm), unexpected reservoirs inside the sample box. Nevertheless, this estimate gives a fundamental frequency of the right order of magnitude.

We estimate the quality factor of the dominant mode in Supplementary Fig. 5b and find $Q \approx 700$ for the $f_0 = 5.6$ Hz peak. We may compare this with the expression from Ref. [7], which is valid for damping of short-wavelength surface waves. Extrapolation of their results to longer wavelengths gives $Q \propto \omega_{\mathrm{He}}/k_{\mathrm{He}} = 1/\sqrt{k_{\mathrm{He}}}$, and thus $Q \sim 10^2 - 10^3$, which agrees with our measured value. Plugging in these values yields an estimated damping time of $\tau \approx 20$ s, which should scale linearly with the reservoir radius r.

We have tried to turn off the pulse tube for as long as 120 seconds before measuring the electron coherence time and even though this is longer than the estimated damping time, we have not observed a decrease in electron linewidth. It is possible that we have underestimated the quality factor, or the electron temperature increased during the period the cooling was stopped. This could occur through heating from the fill lines, even though we did not observe a direct increase in mixing chamber temperature. Further research is needed to fully understand the observed results or alternatively, the experiment may be performed in a wet dilution refrigerator (i.e. a refrigerator without pulse tube cooler).

Supplementary Note 2. Comparison of experimental and modeled unloading voltages

Supplementary Table I. Modeled unloading voltages compared with experimental jump locations

Unloading voltage	Model (V)	Experiment (V)	Comment
$V_{\rm tg}^{(1)}$	-0.305	-0.305	Taken from experiment and used as parameter in the model
$V_{\rm tg}^{(2)}$	-0.248	-0.246	Obtained from fit with $\omega_e/2\pi = 26$ GHz
$V_{\rm tg}^{(3)}$	-0.205	-0.202	Obtained from fit with $\omega_e/2\pi = 26$ GHz
$V_{\rm tg}^{(4)}$	-0.165	-0.168	Obtained from fit with $\omega_e/2\pi = 26$ GHz
$V_{\rm tg}^{(5)}$	-0.127		Not observed in experiment
$V_{\rm tg}^{(6)}$	-0.092		Not observed in experiment

Supplementary Note 3. Orbital frequencies of small electron clusters

For the coefficients α_i that reproduce the data of Fig. 3c in the main text, we plot the eigenfrequencies and electron positions (x_i, y_i) as function of $V_{\rm trap}$ in Supplementary Fig. 6. Only for $N = 1$ the model predicts a crossing of an electron mode with the resonator. For higher N none of the electron mode frequencies cross f_0 over the entire range of simulated $V_{\rm trap}$. Additionally, for $N = 4, 3$ and 2, jumps in frequency and position indicate electron rearrangements initiated by changes in the trap shape. For example, at low $V_{\rm trap}$ two electrons arrange in the across channel direction, whereas at higher $V_{\rm trap}$ it is energetically favorable to arrange in the along-channel direction.

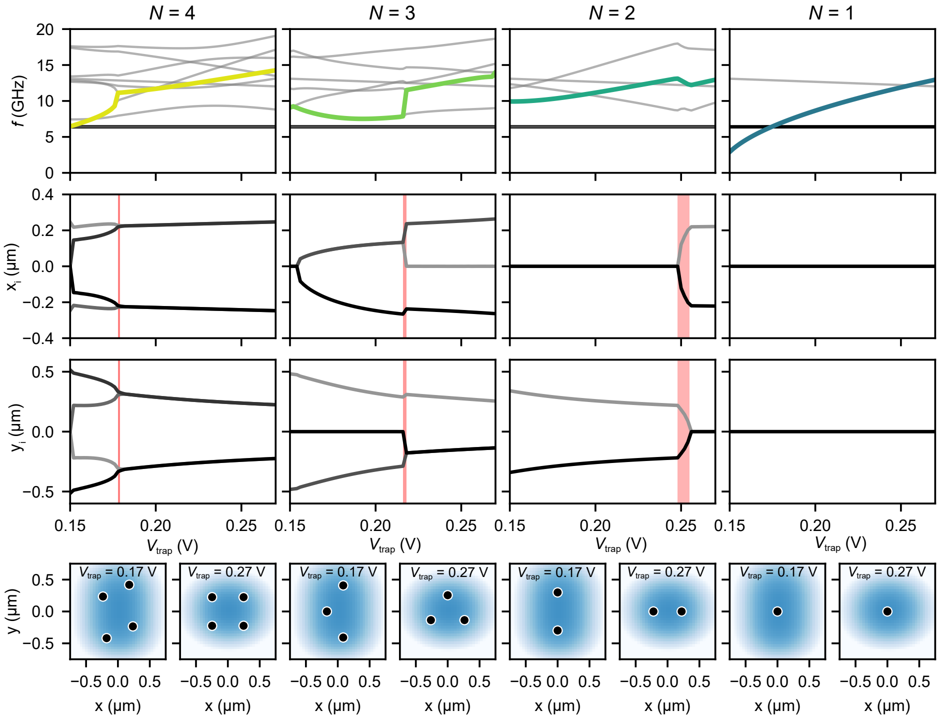

Supplementary Figure 6. Mode frequencies and electron coordinates as function of $V_{\rm trap}$, associated with the solid black lines from Fig. 3c of the main text. In the top row, the strongest coupled electron mode is highlighted and the cavity mode is shown in black. Modes that couple weakly are shown in gray. The two center rows show electron rearrangements within small electron clusters at voltages indicated by the red bands. For each N, snapshots of the electron configuration at $V_{\rm trap} = 0.17$ and 0.27 V further illustrate these rearrangements. The electrostatic potential is shown in shades of blue, with the same colorbar as in Fig. 3d of the main text.

Supplementary Note 4. Simulation of anharmonicity of a single electron

To use a single electron on helium as a qubit, its electrostatic potential needs to be anharmonic. To quantify the anharmonicity of the potential, we solve the Schrödinger equation for a single electron in a two-dimensional electrostatic potential where the spacing of the eigenstates reveals the anharmonicity.

In Supplementary Fig. 7a we plot the transition frequencies from the ground state for the exact same electrode voltages as in Fig. 4 of the main text. The color of each line reflects the calculated coupling strength of each transition, which is calculated from the differential mode amplitude V_{RF} and the ground and excited state wavefunctions. Mathematically it takes on the form

$$g_{0i}/2\pi = \frac{e}{2\pi}\iint \langle 0| \left(x\frac{\partial V_{\mathrm{RF}}}{\partial x} + y\frac{\partial V_{\mathrm{RF}}}{\partial y} \right) |i\rangle dx\, dy, \tag{12}$$

where $i = 0, 1_y, 1_x, 2_y, \ldots$ are the eigenmodes. It is clear that the ground state $|0\rangle$ is most strongly coupled to the first excited state in the y-direction (i.e. $|1_y\rangle$) and the coupling strength reaches several MHz, which is in agreement with the estimate from Supplementary Note 1 B. Direct transitions from the ground state to other states are either forbidden by symmetry (e.g. $|0\rangle \Longleftrightarrow |2_y\rangle$) or extremely weakly coupled due to vanishing electric field (e.g. $|0\rangle \Longleftrightarrow |1_x\rangle$).

Supplementary Fig. 7a further correctly predicts a crossing of the $|0\rangle \Longleftrightarrow |1_y\rangle$ transition with the resonator around $V_{\mathrm{trap}} \approx 0.18$ V. At the crossing, which is indicated by a red star, the sensitivity is $\partial f_e/\partial V_{\mathrm{trap}} \approx 95$ GHz V^{-1}, and the wavefunction of $|1_y\rangle$ is shown in Supplementary Fig. 7c. The next two higher excited states at the crossing voltage are marked with a square and circle (Supplementary Fig. 7d and e, respectively), and we identify those as $|2_y\rangle$ and $|1_x\rangle$. Note the similarity between the wave functions from Supplementary Fig. 7c-f and those of a two-dimensional harmonic oscillator. However, unlike a harmonic oscillator, closer inspection of the transition frequencies reveals that the frequency spacing is non-uniform. In Supplementary Fig. 7g we plot the anharmonicity, defined as the difference between $f_{|1_y\rangle\to|2_y\rangle}$ and $f_{|0\rangle\to|1_y\rangle}$. At the crossing the anharmonicity exceeds 0.1 GHz, indicating that the electron can be approximated as a two-level system.

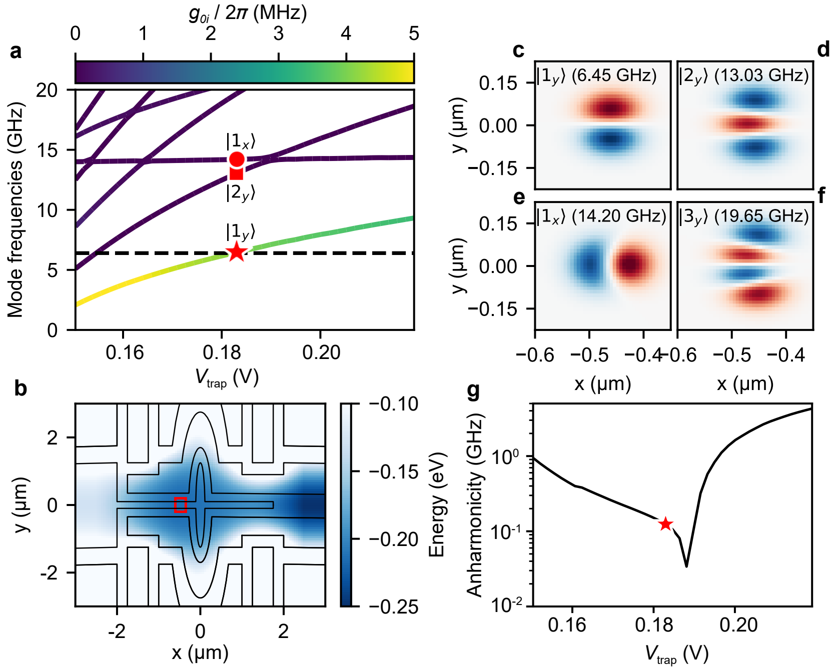

Supplementary Figure 7. Quantum mechanical calculation of a single electron on helium (a) Transition frequencies for a single electron in the ground state, calculated by solving the Schrödinger equation in a two-dimensional electrostatic potential. The the coupling strength g_{0i} for each state $|i\rangle$ is reflected in the color of each line. The resonator frequency is shown as a black dashed line. (b) Simulated electrostatic potential at $z = 1.15$ µm and $V_{\mathrm{trap}} = 0.184$ V. A red rectangle shows the extent of the single-electron wavefunctions shown in (c)-(f). (c)-(f) Real part of the wave functions of the excited states of a single electron near the crossing voltage $V_{\mathrm{trap}} = 0.184$ V. The deep red and blue areas in each plot indicate coordinates where the electron is most likely to be found. (g) Inferred anharmonicity of a single electron as function of trap voltage. A red star marks the anharmonicity at the crossing voltage.

Supplementary Note 5. Single electron response to increased temperature

In many circuit QED experiments temperature is an important parameter which, for example, controls excess photon noise and qubit dephasing. Experiments therefore operate at temperatures such that $k_{\mathrm{B}}T \ll hf_0$ where f_0 is the transition frequency of the resonator or qubit. A natural question is how an electron on helium responds to increased temperature.

In Supplementary Fig. 8 we plot the single electron resonator response as function of temperature. Since bias voltages are equal between traces, and the vibration amplitude of the superfluid helium surface is unaffected by temperature below $T \approx 0.25$ K, the observed broadening of the signal can likely be attributed to heating of the electron. After fitting each trace (keeping g constant between traces), we find that an increased temperature results in an increased linewidth, possibly due to thermal excitations of the orbital state.

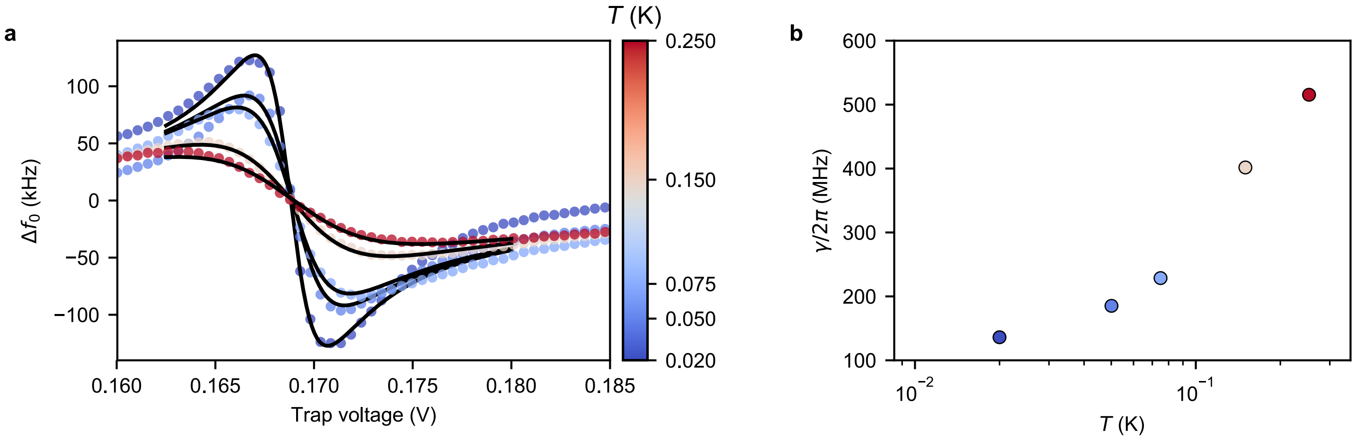

Supplementary Figure 8. Response of a single electron to an increased mixing chamber temperature (a) Single electron resonator spectroscopy traces as function of temperature, from cold ($T = 20$ mK, blue circles) to hot ($T = 250$ mK, red circles). Fits to each dataset are shown as solid black lines. (b) The extracted linewidths from fits to traces in (a) show an increase in linewidth of the orbital state as the mixing chamber temperature increases.

Supplementary Note 6. Contributions to single electron linewidth

In this Supplementary Note we discuss the possible noise sources that contribute to the measured single electron linewidth γ. In general, the linewidth can be written as the sum of the dephasing rate γ_φ, and transverse decay γ_1:

$$\gamma = \frac{\gamma_1}{2} + \gamma_\varphi. \tag{13}$$

An extensive list of decoherence mechanisms for the orbital state of an electron on helium is already available in the supplement of Ref. [8]. Those calculations, which include the polarization of liquid helium, two ripplon decay processes, voltage noise through the electrodes and more, yield that γ_1 and γ_φ should be sub-MHz. Since the observed linewidth is much larger, we consider additional sources of decoherence in the sections below. We list the magnitude of mechanisms and whether they contribute dephasing or decay in Supplementary Table II. In the following section we briefly discuss each mechanism, starting with the dominant cause of dephasing: helium vibrations in the dot area.

Supplementary Table II. Summary of the contributions to the linewidth of a single electron from different types of noise or decay expected in our device

Type	Mechanism	Magnitude
Dephasing	Voltage noise from the gates	0.5 MHz
Dephasing	Helium vibrations in the dot	110 MHz
Dephasing	Reservoir electrons on the resonator	20 MHz
Transverse	Microwave leakage through gates	< 1 MHz

A. Helium vibrations in the dot

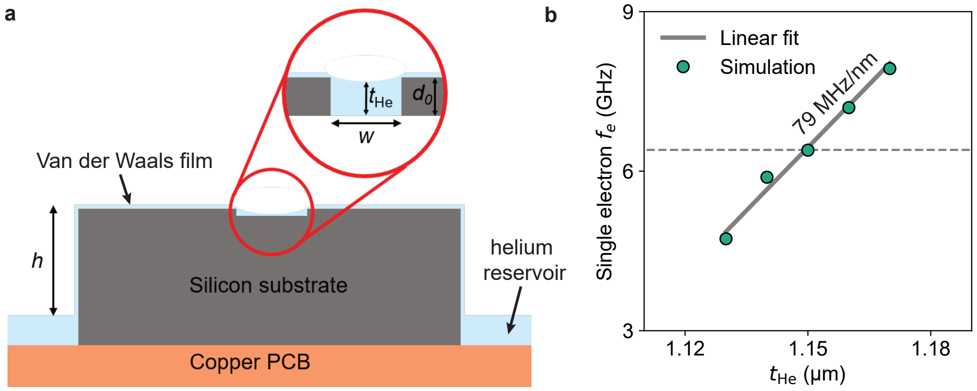

Supplementary Figure 9. Helium vibrations and their effect on a single electron in the dot (a) Schematic of the helium in the channel and the helium in the off-chip reservoir, separated by a distance h. The zoom-in shows a close up of the helium inside the channel. (b) Frequency of a single electron trapped in the dot as function of the helium thickness measured in the center of the channel. For each t_{He}, f_{e} is determined at constant electrode voltages ($V_{\mathrm{trap}} \approx 0.184$ V). A linear fit gives the sensitivity of a single electron w.r.t. t_{He} near the resonator crossing ($f_{\mathrm{e}} = f_0$), which contributes to the linewidth γ. The slope of this line is 79 MHz nm^{-1}.

Since the electrostatic potential varies with the helium thickness t_{He}, helium fluctuations in the dot are a source of dephasing. Helium thickness fluctuations in the micro-channel originate from vibrations in the reservoir, where the helium is not stabilized by surface tension. We can estimate how the magnitude of these vibrations scales with the channel geometry using Jurin's law. In the limit the channel is almost completely filled with helium (see Eq. (10)), the helium height in the center of the channel can be written as

$$t_{\mathrm{He}} = d_0 - \frac{\rho g h w^2}{8\sigma}, \tag{14}$$

where h is the height from the chip to the reservoir level, $\sigma = 3.78 \times 10^{-4}$ N m^{-2} is the surface tension of helium and w is the channel width. Therefore, fluctuations in t_{He} due to level fluctuations inside the off-chip helium reservoir are

given by

$$\Delta t_{\mathrm{He}} = \frac{\partial t_{\mathrm{He}}}{\partial h}\Delta h = \frac{\rho g w^2}{8\sigma}\Delta h \tag{15}$$

Eq. (15) predicts that Δt_{He} scales as w^2, so helium fluctuations are expected to be worse near areas where the channel widens, such as the dot area and the spiral inductor.

From the measurements presented in Supplementary Fig. 5 and a simulated helium-resonator coupling of 5 kHz nm^{-1}, we estimate a magnitude of helium fluctuations of $\Delta t_{\mathrm{He}} \approx 1.4$ nm. With a single electron in the dot, the contribution from helium vibrations to the linewidth is then given by

$$\frac{\gamma_\varphi^{\mathrm{He}}}{2\pi} = \frac{\partial f_e}{\partial t_{\mathrm{He}}}\Delta t_{\mathrm{He}}, \tag{16}$$

where we estimate the electron sensitivity $\partial f_e/\partial t_{\mathrm{He}} = 79$ MHz nm^{-1} (Supplementary Fig. 9b). Finally, we arrive at the contribution due to helium fluctuations in the dot area: $\gamma_\varphi^{\mathrm{He}}/2\pi \approx 110$ MHz.

B. Voltage noise from the gates

Voltage noise on the electrodes in the dot area changes the electrostatic potential and thus leads to dephasing. It is either caused by electrical pickup or Johnson noise. We reduce it by using low-noise voltage sources and filtering the DC lines. Our RC-filters at the mixing chamber plate have a corner frequency of $f_{3\mathrm{dB}} \approx 300$ Hz for the resonator guard, trap guard and trap electrode and $f_{3\mathrm{dB}} \approx 4$ Hz for the resonator electrode.

The linewidth due to voltage noise depends on the electron's sensitivity to each electrode, which we simulate by varying each voltage around the crossing voltage. The slope $\partial f_e/\partial V_{\mathrm{i}}$ at the crossing voltage is a measure of the electron's sensitivity to electrode i. The noise on each electrode adds in quadrature, which leads to a total dephasing rate

$$\frac{\gamma_\varphi^{\mathrm{noise}}}{2\pi} = \sqrt{\sum_i \left(\frac{\partial f_e}{\partial V_i}\right)^2 \Delta V_i^2}. \tag{17}$$

Supplementary Table III lists sensitivities and the total dephasing rate, assuming each electrode has approximately 5 µV of voltage noise. The total estimated contribution due to voltage noise is 0.5 MHz.

Supplementary Table III. Simulated effect of each electrode on the single electron mode frequency f_e and the resulting contribution to the single electron linewidth, assuming a voltage noise on each electrode of 5 µV

Electrode i	Simulated slope (GHz V^{-1})	$\gamma_\varphi^i/2\pi$ (MHz)
Resonator	-48	0.2
Trap	95	0.5
Resonator guard	-7	<0.1
Trap guard	-11	<0.1
Total		0.5

C. Helium vibrations on the resonator

Reservoir electrons above the resonator form a capacitor with the image charges induced in the resonator electrode below. Fluctuations in the helium level Δt_{He} modulate the capacitance C_{res} of the electron reservoir. Since the resonator electrode also extends into the dot area, fluctuating image currents caused by the fluctuating capacitance cause single electron decoherence.

The capacitance of the sheet of electrons above the resonator can be approximated by a parallel plate capacitance: $C_{\mathrm{res}} = \varepsilon_0 \varepsilon_{\mathrm{He}} A / t_{\mathrm{He}}$, where t_{He} is the height of the electrons above the electrode, and A the area of the electron reservoir. A change in the helium thickness causes a change in the capacitance

$$\Delta C_{\mathrm{res}} = -\frac{C_{\mathrm{res}}}{t_{\mathrm{He}}} \Delta t_{\mathrm{He}}. \tag{18}$$

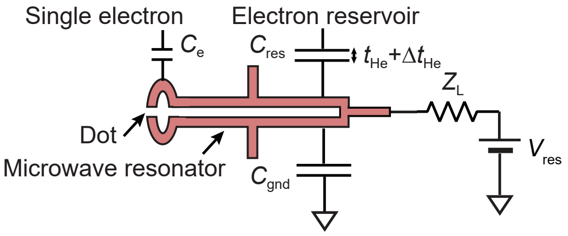

Supplementary Figure 10. Electrical schematic used to calculate the effect of a fluctuating electron reservoir on a single electron. The reservoir is representated by the capacitor C_{res}, which fluctuates due to classical helium fluctuations Δt_{He}. The resonator electrode is biased using a voltage source, which is hooked up to the electrode with leads that have impedance Z_L. This impedance also includes the bias source output impedance and filters.

The resonator is biased with a voltage source and its output impedance and line impedance are parametrized by Z_L, as shown in Supplementary Fig. 10. The effect of ΔC_{res} on a single electron depends on the value of Z_L. If $Z_L \to \infty$, the resonator potential is floating and fluctuating image currents do not dissipate via the leads but are dissipated via the capacitors. An estimate of the single-electron linewidth assuming $Z_L \to \infty$ therefore represents an upper dephasing limit. Below we derive such an estimate, which further assumes that the helium thickness fluctuations are quasistatic, as can be verified from the vibration spectrum in Supplementary Fig. 5.

If the resonator is electrically floating, a small fluctuation in capacitance results in voltage noise on the resonator electrode via $V = Q/C_{\mathrm{tot}}$, such that the voltage noise ΔV_{res} is given by:

$$\Delta V_{\mathrm{res}} = -\frac{V_{\mathrm{res}}}{C_{\mathrm{tot}}} \Delta C_{\mathrm{res}} = \frac{V_{\mathrm{res}}}{t_{\mathrm{He}}} \frac{C_{\mathrm{res}}}{C_{\mathrm{tot}}} \Delta t_{\mathrm{He}}. \tag{19}$$

In Eq. (19) we define the total capacitance of the resonator electrode $C_{\mathrm{tot}} \approx C_{\mathrm{res}} + C_{\mathrm{gnd}}$ and have assumed that (i) the capacitance to the single electron C_e is negligible and (ii) C_{gnd} is approximately constant with t_{He}. The ratio $C_{\mathrm{res}}/C_{\mathrm{tot}}$ equals the resonator electrode lever arm at the location of the electron reservoir, and is approximately 0.6 from electrostatic simulations.

Finally, using Eq. (19), we estimate the dephasing from the electron sensitivity to the resonator electrode:

$$\frac{\gamma_\varphi^{\mathrm{res}}}{2\pi} = \left| \frac{\partial f_e}{\partial V_{\mathrm{res}}} \right| \Delta V_{\mathrm{res}} = \left| \frac{\partial f_e}{\partial V_{\mathrm{res}}} \right| \frac{V_{\mathrm{res}}}{t_{\mathrm{He}}} \frac{C_{\mathrm{res}}}{C_{\mathrm{tot}}} \Delta t_{\mathrm{He}} \tag{20}$$

Assuming $\Delta t_{\mathrm{He}} = 1.4$ nm, $t_{\mathrm{He}} = 1.2$ µm, $V_{\mathrm{res}} = 0.6$ and $\partial f_e / \partial V_{\mathrm{res}}$ from Supplementary Table III, we estimate an upper limit for the single-electron linewidth $\gamma_\varphi^{\mathrm{res}}/2\pi \approx 20$ MHz. We assume there are no electrons on the trap, resonator guards or trap guards, such that a similar calculation for these electrodes does not result in additional dephasing. In future devices, this source of dephasing can be eliminated completely by removing the reservoir electrons or using an additional reservoir that does not couple to the resonator.

D. Microwave leakage through DC bias electrodes

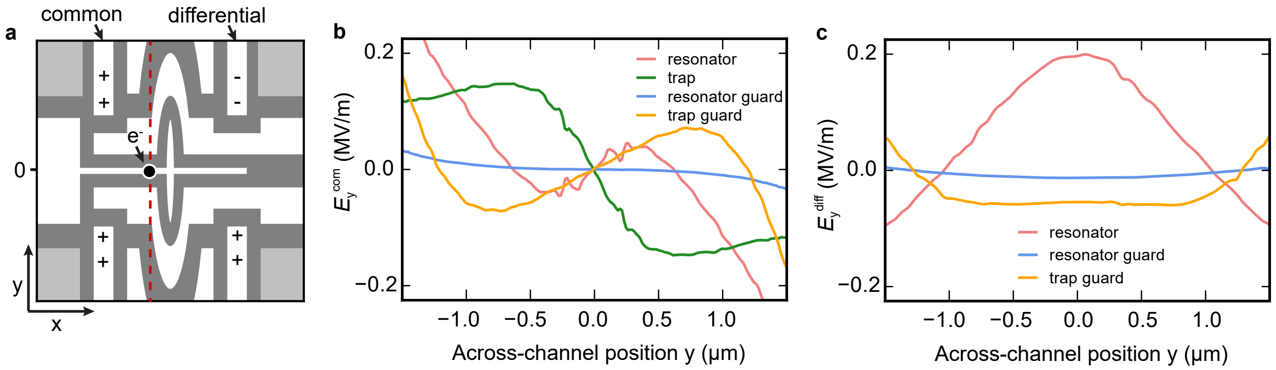

Supplementary Figure 11. Estimating decay through DC bias electrodes (a) Schematic of the electrode geometry in the dot area, showing the expected electron position and an example of charge distributions on the trap guard and resonator guard which correspond to common and differential microwave leakage, respectively. (b) Common mode E_y simulated at the red dashed line in (a). The values of E_y at the electron position provide an estimate for microwave leakage through each respective electrode. (c) Same as in (b), but for the differential mode. Note that the trap electrode does not support a differential mode.

Ideally, the resonator is the only electrode that couples to the electron's motion and DC bias electrodes form perfect microwave reflectors from the electron's perspective. In practice there is always some leakage, even though we have taken the following measures to reduce this unwanted effect:

- Adding a low-pass LC-filter on each DC bias electrode, and
- Shorting the left and right electrodes of each pair of guard electrodes.

To quantify microwave leakage, we note that the coupling strength is set by $g = \mathbf{d} \cdot \mathbf{E}$. The electric field of each electrode in the across-channel direction therefore determines the leakage (i.e. coupling). Except for the trap electrode, we model microwave emission into each electrode by considering a common and differential mode, which are shown in Supplementary Fig. 11a, b. Since the electron couples to the differential mode of the resonator with $g/2\pi \approx 5$ MHz, and all other electric fields at the electron position are much smaller than E_y^{diff} of the resonator, we estimate the total decay from leakage through the bias electrodes to be $\gamma/2\pi < 1$ MHz.

Supplementary Note 7. Signs of helium vibrations in the crossing spectrum

Here we present extra evidence to support our claim that the single-electron linewidth is significantly affected by helium fluctuations. Since helium fluctuations change the single-electron orbital frequency, its crossing voltage with the resonator is expected to vary with time. We attempted to measure this effect by repeatedly bringing the orbital frequency into resonance using the trap electrode. The JPA ensures a high signal-to-noise ratio, such that we can accurately estimate the crossing voltage by fitting the normalized transmission during a single voltage ramp (see Supplementary Fig. 12b). After repeating the experiment 10^4 times, we obtain an average crossing voltage of $V_{\text{trap}} \approx 0.1805$ V with a standard deviation of $\Delta V_{\text{trap}} = 0.3$ mV. Using the simulated sensitivity of the trap electrode of $\partial f_e / \partial V_{\text{trap}} = 95$ GHz V^{-1}, this spread in the crossing voltage corresponds to a single electron linewidth

$$\gamma/2\pi = 2\sqrt{2\ln 2}\frac{\partial f_e}{\partial V_{\text{trap}}}\Delta V_{\text{trap}} = 67\,\text{MHz}, \tag{21}$$

which agrees with the value from the main text: $\gamma_\varphi/2\pi = (77 \pm 19)$ MHz.

Since the time of each crossing is known from the ramp, we can Fourier transform the crossing time series (red dots in Supplementary Fig. 12a) to learn about the spectral content. The spectrum of the crossing voltage shows distinct peaks at even multiples of the pulse tube refrigerator (1.4 Hz) and looks very similar to the bare helium fluctuation spectrum measured in Supplementary Fig. 5b. Therefore, these data directly show the effect of helium vibrations on a single electron.

We have attempted to refocus individual crossings from Supplementary Fig. 5b in post-processing but did not observe an increase in linewidth after fitting the averaged refocused data. It is possible that helium vibrations with frequencies larger than 12 Hz still contribute significantly to the spectrum. The maximum frequency we can detect in the crossing spectrum is limited by the repetition rate of the experiment ($f_{\text{rep}} \approx 22.2$ Hz). For this experiment the corner frequency of the RC-filters prevented measurement of higher frequency components in the spectrum. However, by removing these filters this technique could be used to characterize the spectral density of a single electron even at higher frequencies.

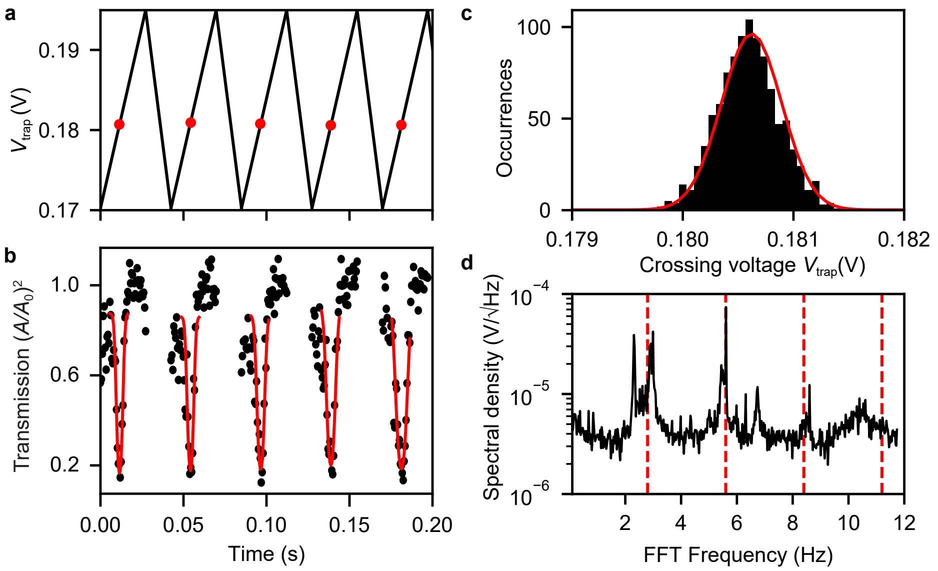

Supplementary Figure 12. Statistics on fast single electron sweeps. (a) After loading a single electron we sweep the trap over a range of 25 mV while measuring the resonator transmission. A red dot marks the determined crossing voltage found by fitting the signal displayed in (b). (b) The resonator transmission shows unaveraged crossings of a single electron with the resonator (black dots). The solid red lines are fits which give the time and voltage of the crossing. (c) Statistics of 10^4 crossings with the resonator. The solid red line is a Gaussian fit with standard deviation $\Delta V_{\text{trap}} = 0.28$ mV. (d) Fourier transform (FFT) of the crossing voltage time series. The dashed lines at 2.8, 5.6, 8.4 and 11.2 Hz indicate multiples of the pulse tube refrigerator frequency, and align with peaks in the crossing spectrum.

Supplementary References

[1] Yang, G. *et al.* Coupling an ensemble of electrons on superfluid helium to a superconducting circuit. *Phys. Rev. X* **6**, 011031 (2016).

[2] Mueller, F. *et al.* Printed circuit board metal powder filters for low electron temperatures. *Review of Scientific Instruments* **84**, 044706 (2013).

[3] Mi, X. *et al.* Circuit quantum electrodynamics architecture for gate-defined quantum dots in silicon. *Applied Physics Letters* **110**, 043502 (2017).

[4] Kashkanova, A. D. *et al.* Superfluid brillouin optomechanics. *Nature Physics* **13**, 74–79 (2016). Article.

[5] Harris, G. I. *et al.* Laser cooling and control of excitations in superfluid helium. *Nature Physics* **12**, 788–793 (2016). Article.

[6] Lorenzo, L. A. D. & Schwab, K. C. Superfluid optomechanics: coupling of a superfluid to a superconducting condensate. *New Journal of Physics* **16**, 113020 (2014).

[7] Roche, P., Deville, G., Keshishev, K. O., Appleyard, N. J. & Williams, F. I. B. Low damping of micron capillary waves on superfluid ^{4}he. *Phys. Rev. Lett.* **75**, 3316–3319 (1995).

[8] Schuster, D. I., Fragner, A., Dykman, M. I., Lyon, S. A. & Schoelkopf, R. J. Proposal for manipulating and detecting spin and orbital states of trapped electrons on helium using cavity quantum electrodynamics. *Phys. Rev. Lett.* **105**, 040503 (2010).
